# Haploinsufficiency Interactions of RALBP1 and TP53 in Carcinogenesis

**DOI:** 10.3390/cancers13020255

**Published:** 2021-01-12

**Authors:** Sanjay Awasthi

**Affiliations:** Division of Hematology & Oncology, Texas Tech Health Sciences Center 3601 4th St., Lubbock, TX 79430-9410, USA; Sanjay.Awasthi@ttuhsc.edu

Mutagenic environmental chemical or radiant stresses directly damage DNA and amplify the formation of potent endogenous mutagens from lipid peroxidation, leading to cancers that cause millions of deaths and impose enormous financial and social burdens [1,2]. The p53 protein (encoded by the *TP53* gene (*TP53*) and used here for both gene and protein), has been dubbed the ‘Guardian of the Genome’ because of its pleiotropic genoprotective functions. Germline mutations at ‘hotspot’ regions of the *TP53* gene strongly predispose cancer in persons with Li–Fraumeni syndrome and somatic mutations of *TP53* are found in the majority of cancers [3,4,5,6,7,8,9]. Nearly all mice carrying homozygous *TP53* deletions die of spontaneous cancer before reaching 6 months of age. In stark contrast, mice lacking the *RALBP1* ((RalA Binding Protein 1) gene, which encodes RLIP76 (also known as Rlip (RALBP1), used here for both gene and protein), a stress-responsive, anti-apoptotic protein, are highly resistant to carcinogenesis by even the most potent chemical carcinogens [10]. Targeted inhibition or depletion of RALBP1 causes regression of many types of cancer [10,11,12,13,14,15,16]. In studies to examine the effect of combined RALBP1 and TP53 deficiency, we discovered that hemizygous RALBP1 deficiency was sufficient to exert a strong dominant negative effect on the spontaneous carcinogenesis phenotype of homozygous TP53-null mice [10]. The studies presented here provide the first evidence for RALBP1 haploinsufficiency in the prevention of *ERBB2* (Erb-B2 Receptor Tyrosine Kinase 2) -driven breast cancer. Additionally, the molecular mechanisms through which RALBP1 may exert transcriptional regulation and DNA repair and the effects of Ω-6 fatty acid metabolites in cancer are explored here by a spectrum of researchers who have made important contributions to defining the pleiotropic molecular mechanisms that have begun to define a new paradigm in carcinogenesis.

RALBP1 is an ATPase enzyme of the mercapturic acid pathway that catalyzes the transmembrane efflux of Glutathione (GSH)-electrophile thioether conjugates (GS-E) that are formed through the glutathione S-transferase (GST)-catalyzed conjugation of GSH with electrophilic toxins [17,18,19,20,21,22,23,24,25,26,27,28,29,30,31,32,33,34,35,36,37]. Its ATPase activity is coupled with clathrin-dependent endocytosis (CDE), the RAL-regulated first step in the internalization and trafficking of membrane vesicles containing receptor-bound, cancer-promoting growth hormones [38,39]. CDE regulates the downstream signaling of receptors for insulin, EGF, TNFα, FGF1 and many other peptide hormones [40,41,42,43]. Concomitant deficiencies of GS-E efflux, GSH-linked antioxidant defenses and CDE-linked vesicular trafficking in RALBP1-null (RALBP1^−/−^) mice show that RALBP1 functions provide an essential link between stress-induced apoptosis defenses and RAL/RAS/RHO/RAC-regulated pathways that promote cancer cell growth [44,45,46,47,48,49,50,51]. Our recent studies in lung and breast cancers as well as melanoma have confirmed that the inhibition or depletion of RALBP1 blocks CDE and inhibits signaling by cancer-promoting peptides, including EGF, WNT and FGF [10,11,13,14]. The role of RALBP1 in regulating intracellular vesicular traffic, and the signaling proteins, such as ARF6, ARNO, PI3K and RAC, that regulate vesicular traffic, is reviewed in one paper presented in this symposium issue. Furthermore, the X-ray crystallographic elucidation of the RALBP1 structure, which sheds much-needed light on the molecular mechanisms for these pleiotropic effects, is presented.

Because GS-E conjugates are potent inhibitors of upstream xenobiotic metabolizing enzymes and GSH-linked antioxidant defenses, GS-E accumulation caused by RALBP1 deficiency profoundly impairs the ability of highly metabolically active cancer cells to defend against apoptosis caused by exogenous as well as endogenously generated toxins. Oxidative metabolism of Ω-6 polyunsaturated fatty acids triggered by radiant (X-ray, UV light and heat) or oxidative stress yields lipid hydroperoxides that degrade to toxic lipid alkenals, principally 4-hydroxynonenal (4HNE). The highly reactive alkylating agent 4HNE, with inherently greater pro-apoptotic effects against malignant as compared with non-malignant cells, is metabolized primarily to a glutathione-conjugate (GS-HNE) that is removed from cells by RALBP1 [18,26,27,36,37,52,53,54]. Elevated 4HNE production from the high levels of oxidative stress imposed on cancer cells by their high metabolic rates renders them very dependent on this mechanism of metabolizing and disposing of 4HNE, as is evident from the dramatic and sustained regression of multiple histological types of cancer upon depletion or inhibition of RALBP1 in mouse models without any toxicity [10,11,55]. Our findings of inhibition of cancer growth in vitro and in vivo have been confirmed by several independent groups [44,48,49,56,57,58]. Evidence for the role of RALBP1 and other aldehyde-metabolizing enzymes in regulating the cancer-selective apoptotic effects of Ω-6 fatty acids and 4HNE in breast cancer is presented in one of the papers in this symposium.

The inability of grafted mouse melanoma and lung cancer cells to grow in RALBP1^−/−^ mice [49] and the resistance of RALBP1^−/−^ mice to benzo(a)pyrene, phorbol ester or dimethylbenzanthracene-induced carcinogenesis led us test the possibility that RALBP1 has existential importance for cancer cell formation and survival [10]. Because the carcinogenic effects of TP53 loss would be opposed by the reduced survival of malignant or pre-malignant cells due to RALBP1 deficiency, we posited that RALBP1 deficiency would proportionately reduce carcinogenesis in TP53^−/−^ mice. We found that weekly injections of an RALBP1-directed phosphorothioate antisense molecule (R508) to TP53^−/−^ mice starting at 8 weeks of age reduced RALBP1 protein content to half of normal levels and provided complete protection from carcinogenesis. Upon crossing TP53^+/−^ with RALBP1^+/−^ mice, we found a profound reduction in cancer incidence in mice lacking one or two RALBP1 alleles, regardless of TP53 status, and that loss of one RALBP1 allele was sufficient for protection from spontaneous carcinogenesis. Susceptibility to benzo(a)pyrene-induced carcinogenesis was reduced to half in TP53^+/−^RALBP1^+/−^ mice and to one fifth in TP53^+/−^RALBP1^−/−^ mice [10]. The results of our subsequent studies showing distinct cancer-preventative effects of heterozygous vs. homozygous RALBP1 deficiency in ERBB2-overexpression-driven as well as PYVT (Polyoma Virus middle T antigen) -driven breast cancer in TP53^+/−^ mice, presented by Singh et al. in this symposium, strongly support complex haploinsufficiency interactions underlying cancer prevention by RALBP1 deficiency. Previously reported haploinsufficiency interactions of TP53 binding partners, such as MDM2, MDM4, and HSF1, manifest as embryonic lethality in carriers of imbalanced deletions and switches in cancer histological type but do not substantially reduce cancer susceptibility. Concomitant mutations in oncogenes increase the intrinsic cancer susceptibility of TP53^−/−^ mice, but genetic manipulation of single genes has not been shown to suppress carcinogenesis as effectively as RALBP1 haploinsufficiency [59,60,61,62,63,64,65,66,67,68,69,70,71,72,73,74,75,76].

The disproportionate effect on cancer protection correlated with reversion of the marked transcriptomic and promoter methylomic abnormalities in control TP53^−/−^ mice to nearly wild-type in R508-treated TP53^−/−^ mice, in whom RALBP1 was reduced by ≤50%. Whole-genome bisulfite sequencing (WGBS) showed that control TP53^−/−^ mice had over 14,000 differentially methylated regions (DMRs) while R508-treated mice had less than 1000. The prevention of promoter DMRs was associated with prevention of differential expression in over 1600 genes in liver samples by RNA-Seq [10]. Detailed analyses of the transcriptomic effects of congenital RALBP1 deficiency without or with TP53 deletion with respect to age and gender are presented in this symposium issue.

Because the RALBP1-TP53 dimer cannot exist in either the TP53^−/−^ or RALBP1^−/−^ mouse, this complex by itself cannot explain the underlying mechanism. Instead, at least one other protein that binds both RALBP1 and TP53 would be required for an explanation. Such proteins include heat shock factor 1 (Hsf1), cdc2/cdk1, Hsp90, and PKCα. The best-studied of these is Hsf1, the master transcription factor for the chaperone response to heat shock [77] that binds RALBP1 in reciprocal inhibitory interactions [78] and binds TP53 to determine embryonic lethality as well as cancer histology [10,78]. Intriguing new results presented in the symposium on transcriptional regulation of PKCα provide an important insight into the pleotropic mechanisms through which RALBP1 could influence carcinogenesis.

Adult-onset diabetes, obesity, and metabolic syndrome are diseases characterized by insulin resistance (IR) [79] and are now strongly linked with carcinogenic risk [80]. CDE is the primary mechanism for insulin endocytosis and is activated by stressors known to exacerbate IR [38]. Despite having high levels of oxidative stress, RALBP1^+/−^ mice are extraordinarily sensitive to insulin, having low blood glucose as well as lipids [81]. This topic is reviewed in the symposium. Agents that reduce RALBP1 expression or inhibit its activity could reduce cancer risk. In this issue, we present experimental evidence for cancer prevention by one such compound, 2-hydroxyflavanone, which has been shown, by us, to inhibit melanoma as well as lung, breast, and kidney cancers. This compound has served as a lead in developing the novel synthetic RALBP1 inhibitors that are discussed in one of the papers presented in this symposium. Finally, it should be noted that diabetic and obese individuals are at particularly high risk for COVID-19, a virus that enters cells through CDE. Hindle et al. present intriguing indirect evidence for a potential role of RALBP1 in the pathogenesis of this infectious disease.

In summary, we have observed a striking anticancer effect of RALBP1 that is powerful enough to overcome the universally cancer-susceptible phenotype of TP53-null mice, requiring only partial inhibition. This is in stark contrast to other targeted therapies that generally rely on abrogating the target activity, suggesting that RALBP1-targeted drugs could have lower normal tissue toxicity and fewer side effects. The complexity of the molecular mechanisms underlying the cancer-preventative effects of RALBP1 inhibition is not understood. The purpose of this symposium is to assemble a diverse array of scientific research that will collectively elucidate the molecular mechanism for this new paradigm in cancer prevention.

## References

[1] Jemal A., Bray F., Center M.M., Ferlay J., Ward E., Forman D. (2011). Global cancer statistics. CA Cancer J. Clin..

[2] Mariotto A.B., Yabroff K.R., Shao Y., Feuer E.J., Brown M.L. (2011). Projections of the cost of cancer care in the United States: 2010–2020. J. Natl. Cancer Inst..

[3] George J., Lim J.S., Jang S.J., Cun Y., Ozretic L., Kong G., Leenders F., Lu X., Fernandez-Cuesta L., Bosco G. (2015). Comprehensive genomic profiles of small cell lung cancer. Nature.

[4] Cancer Genome Atlas Research Network (2012). Comprehensive genomic characterization of squamous cell lung cancers. Nature.

[5] Jordan E.J., Kim H.R., Arcila M.E., Barron D., Chakravarty D., Gao J., Chang M.T., Ni A., Kundra R., Jonsson P. (2017). Prospective Comprehensive Molecular Characterization of Lung Adenocarcinomas for Efficient Patient Matching to Approved and Emerging Therapies. Cancer Discov..

[6] Bieging K.T., Mello S.S., Attardi L.D. (2014). Unravelling mechanisms of p53-mediated tumour suppression. Nat. Rev. Cancer.

[7] Chen P.L., Chen Y.M., Bookstein R., Lee W.H. (1990). Genetic mechanisms of tumor suppression by the human p53 gene. Science.

[8] Levine A.J. (1997). p53, the cellular gatekeeper for growth and division. Cell.

[9] Ariffin H., Hainaut P., Puzio-Kuter A., Choong S.S., Chan A.S., Tolkunov D., Rajagopal G., Kang W., Lim L.L., Krishnan S. (2014). Whole-genome sequencing analysis of phenotypic heterogeneity and anticipation in Li-Fraumeni cancer predisposition syndrome. Proc. Natl. Acad. Sci. USA.

[10] Awasthi S., Tompkins J., Singhal J., Riggs A.D., Yadav S., Wu X., Singh S., Warden C., Liu Z., Wang J. (2018). Rlip depletion prevents spontaneous neoplasia in TP53 null mice. Proc. Natl. Acad. Sci. USA.

[11] Bose C., Yadav S., Singhal S.S., Singhal J., Hindle A., Lee J., Cheedella N.K.S., Rehman S., Rahman R.L., Jones C. (2020). Rlip Depletion Suppresses Growth of Breast Cancer. Cancers.

[12] Singhal J., Chikara S., Horne D., Salgia R., Awasthi S., Singhal S.S. (2019). RLIP inhibition suppresses breast-to-lung metastasis. Cancer Lett..

[13] Bose C., Singh S.P., Igid H., Green W.C., Singhal S.S., Lee J., Palade P.T., Rajan A., Ball S., Tonk V. (2019). Topical 2′-Hydroxyflavanone for Cutaneous Melanoma. Cancers.

[14] Awasthi S., Singhal S.S., Singhal J., Nagaprashantha L., Li H., Yuan Y.C., Liu Z., Berz D., Igid H., Green W.C. (2018). Anticancer activity of 2’-hydroxyflavanone towards lung cancer. Oncotarget.

[15] Singhal S.S., Nagaprashantha L., Singhal P., Singhal S., Singhal J., Awasthi S., Horne D. (2017). RLIP76 Inhibition: A promising developmental therapy for neuroblastoma. Pharm. Res..

[16] Singhal S.S., Singhal J., Figarola J., Horne D., Awasthi S. (2015). RLIP76 Targeted Therapy for Kidney Cancer. Pharm. Res..

[17] Awasthi S., Cheng J., Singhal S.S., Saini M.K., Pandya U., Pikula S., Bandorowicz-Pikula J., Singh S.V., Zimniak P., Awasthi Y.C. (2000). Novel function of human RLIP76: ATP-dependent transport of glutathione conjugates and doxorubicin. Biochemistry.

[18] Awasthi S., Cheng J.Z., Singhal S.S., Pandya U., Sharma R., Singh S.V., Zimniak P., Awasthi Y.C. (2001). Functional reassembly of ATP-dependent xenobiotic transport by the N- and C-terminal domains of RLIP76 and identification of ATP binding sequences. Biochemistry.

[19] Awasthi S., Singhal S.S., Pikula S., Piper J.T., Srivastava S.K., Torman R.T., Bandorowicz-Pikula J., Lin J.T., Singh S.V., Zimniak P. (1998). ATP-Dependent human erythrocyte glutathione-conjugate transporter. II. Functional reconstitution of transport activity. Biochemistry.

[20] Awasthi S., Singhal S.S., Srivastava S.K., Torman R.T., Zimniak P., Bandorowicz-Pikula J., Singh S.V., Piper J.T., Awasthi Y.C., Pikula S. (1998). ATP-Dependent human erythrocyte glutathione-conjugate transporter. I. Purification, photoaffinity labeling, and kinetic characteristics of ATPase activity. Biochemistry.

[21] Awasthi Y.C., Sharma R., Singhal S.S. (1994). Human glutathione S-transferases. Int. J. Biochem..

[22] Awasthi S., Singhal S.S., Yadav S., Singhal J., Drake K., Nadkar A., Zajac E., Wickramarachchi D., Rowe N., Yacoub A. (2005). RLIP76 is a major determinant of radiation sensitivity. Cancer Res..

[23] Awasthi S., Singhal S.S., Yadav S., Singhal J., Vatsyayan R., Zajac E., Luchowski R., Borvak J., Gryczynski K., Awasthi Y.C. (2010). A central role of RLIP76 in regulation of glycemic control. Diabetes.

[24] Cheng J.Z., Yang Y., Singh S.P., Singhal S.S., Awasthi S., Pan S.S., Singh S.V., Zimniak P., Awasthi Y.C. (2001). Two distinct 4-hydroxynonenal metabolizing glutathione S-transferase isozymes are differentially expressed in human tissues. Biochem. Biophys. Res. Commun..

[25] Leake K., Singhal J., Nagaprashantha L., Awasthi S., Singhal S.S. (2012). RLIP76 Regulates PI3K/Akt Signaling and Chemo-Radiotherapy Resistance in Pancreatic Cancer. PLoS ONE.

[26] Sharma R., Singhal S.S., Wickramarachchi D., Awasthi Y.C., Awasthi S. (2004). RLIP76 (RALBP1)-mediated transport of leukotriene C4 (LTC4) in cancer cells: Implications in drug resistance. Int. J. Cancer.

[27] Awasthi S., Singhal S.S., Awasthi Y.C., Martin B., Woo J.H., Cunningham C.C., Frankel A.E. (2008). RLIP76 and Cancer. Clin. Cancer Res..

[28] Nagaprashantha L., Vartak N., Awasthi S., Awasthi S., Singhal S.S. (2012). Novel anti-cancer compounds for developing combinatorial therapies to target anoikis-resistant tumors. Pharm. Res..

[29] Drake K.J., Singhal J., Yadav S., Nadkar A., Pungaliya C., Singhal S.S., Awasthi S. (2007). RALBP1/RLIP76 mediates multidrug resistance. Int. J. Oncol..

[30] Figarola J.L., Singhal P., Rahbar S., Gugiu B.G., Awasthi S., Singhal S.S. (2013). COH-SR4 reduces body weight, improves glycemic control and prevents hepatic steatosis in high fat diet-induced obese mice. PLoS ONE.

[31] Singhal S.S., Singhal J., Yadav S., Dwivedi S., Boor P.J., Awasthi Y.C., Awasthi S. (2007). Regression of lung and colon cancer xenografts by depleting or inhibiting RLIP76 (Ral-binding protein 1). Cancer Res..

[32] Singhal S.S., Singhal J., Yadav S., Sahu M., Awasthi Y.C., Awasthi S. (2009). RLIP76: A target for kidney cancer therapy. Cancer Res..

[33] Singhal S.S., Wickramarachchi D., Yadav S., Singhal J., Leake K., Vatsyayan R., Chaudhary P., Lelsani P., Suzuki S., Yang S. (2011). Glutathione-conjugate transport by RLIP76 is required for clathrin-dependent endocytosis and chemical carcinogenesis. Mol. Cancer Ther..

[34] Singhal S.S., Yadav S., Drake K., Singhal J., Awasthi S. (2008). Hsf-1 and POB1 induce drug sensitivity and apoptosis by inhibiting Ralbp1. J. Biol. Chem..

[35] Stuckler D., Singhal J., Singhal S.S., Yadav S., Awasthi Y.C., Awasthi S. (2005). RLIP76 transports vinorelbine and mediates drug resistance in non-small cell lung cancer. Cancer Res..

[36] Yadav S., Singhal S.S., Singhal J., Wickramarachchi D., Knutson E., Albrecht T.B., Awasthi Y.C., Awasthi S. (2004). Identification of membrane-anchoring domains of RLIP76 using deletion mutant analyses. Biochemistry.

[37] Yang Y., Sharma A., Sharma R., Patrick B., Singhal S.S., Zimniak P., Awasthi S., Awasthi Y.C. (2003). Cells preconditioned with mild, transient UVA irradiation acquire resistance to oxidative stress and UVA-induced apoptosis: Role of 4-hydroxynonenal in UVA-mediated signaling for apoptosis. J. Biol. Chem..

[38] Brown M.S., Goldstein J.L. (1979). Receptor-mediated endocytosis: Insights from the lipoprotein receptor system. Proc. Natl. Acad. Sci. USA.

[39] Sorkin A., von Zastrow M. (2009). Endocytosis and signalling: Intertwining molecular networks. Nat. Rev. Mol. Cell Biol..

[40] Jean S., Mikryukov A., Tremblay M.G., Baril J., Guillou F., Bellenfant S., Moss T. (2010). Extended-synaptotagmin-2 mediates FGF receptor endocytosis and ERK activation in vivo. Dev. Cell.

[41] Pinilla-Macua I., Sorkin A. (2015). Methods to study endocytic trafficking of the EGF receptor. Methods Cell Biol..

[42] Singhal S.S., Sehrawat A., Sahu M., Singhal P., Vatsyayan R., Rao Lelsani P.C., Yadav S., Awasthi S. (2010). Rlip76 transports sunitinib and sorafenib and mediates drug resistance in kidney cancer. Int. J. Cancer.

[43] Tsujimoto M., Yip Y.K., Vilcek J. (1985). Tumor necrosis factor: Specific binding and internalization in sensitive and resistant cells. Proc. Natl. Acad. Sci. USA.

[44] Fillatre J., Delacour D., Van Hove L., Bagarre T., Houssin N., Soulika M., Veitia R.A., Moreau J. (2012). Dynamics of the subcellular localization of RalBP1/RLIP through the cell cycle: The role of targeting signals and of protein-protein interactions. FASEB J. Off. Publ. Fed. Am. Soc. Exp. Biol..

[45] Hinoi T., Kishida S., Koyama S., Ikeda M., Matsuura Y., Kikuchi A. (1996). Post-translational modifications of Ras and Ral are important for the action of Ral GDP dissociation stimulator. J. Biol. Chem..

[46] Jullien-Flores V., Mahe Y., Mirey G., Leprince C., Meunier-Bisceuil B., Sorkin A., Camonis J.H. (2000). RLIP76, an effector of the GTPase Ral, interacts with the AP2 complex: Involvement of the Ral pathway in receptor endocytosis. J. Cell Sci..

[47] Kashatus D.F., Lim K.H., Brady D.C., Pershing N.L., Cox A.D., Counter C.M. (2011). RALA and RALBP1 regulate mitochondrial fission at mitosis. Nat. Cell Biol..

[48] Lee S., Goldfinger L.E. (2014). RLIP76 regulates HIF-1 activity, VEGF expression and secretion in tumor cells, and secretome transactivation of endothelial cells. FASEB J. Off. Publ. Fed. Am. Soc. Exp. Biol..

[49] Lee S., Wurtzel J.G., Singhal S.S., Awasthi S., Goldfinger L.E. (2012). RALBP1/RLIP76 depletion in mice suppresses tumor growth by inhibiting tumor neovascularization. Cancer Res..

[50] Rosse C., L’Hoste S., Offner N., Picard A., Camonis J. (2003). RLIP, an effector of the Ral GTPases, is a platform for Cdk1 to phosphorylate epsin during the switch off of endocytosis in mitosis. J. Biol. Chem..

[51] Wurtzel J.G., Lee S., Singhal S.S., Awasthi S., Ginsberg M.H., Goldfinger L.E. (2015). RLIP76 regulates Arf6-dependent cell spreading and migration by linking ARNO with activated R-Ras at recycling endosomes. Biochem. Biophys. Res. Commun..

[52] Zheng R., Po I., Mishin V., Black A.T., Heck D.E., Laskin D.L., Sinko P.J., Gerecke D.R., Gordon M.K., Laskin J.D. (2013). The generation of 4-hydroxynonenal, an electrophilic lipid peroxidation end product, in rabbit cornea organ cultures treated with UVB light and nitrogen mustard. Toxicol. Appl. Pharmacol..

[53] Prasanna P.G., Narayanan D., Hallett K., Bernhard E.J., Ahmed M.M., Evans G., Vikram B., Weingarten M., Coleman C.N. (2015). Radioprotectors and Radiomitigators for Improving Radiation Therapy: The Small Business Innovation Research (SBIR) Gateway for Accelerating Clinical Translation. Radiat. Res..

[54] Sharma R., Singhal S.S., Cheng J., Yang Y., Sharma A., Zimniak P., Awasthi S., Awasthi Y.C. (2001). RLIP76 is the major ATP-dependent transporter of glutathione-conjugates and doxorubicin in human erythrocytes. Arch. Biochem. Biophys..

[55] Singhal S.S., Salgia R., Verma N., Horne D., Awasthi S. (2020). RLIP controls receptor-ligand signaling by regulating clathrin-dependent endocytosis. Biochim. Biophys. Acta Rev. Cancer.

[56] Oxford G., Owens C.R., Titus B.J., Foreman T.L., Herlevsen M.C., Smith S.C., Theodorescu D. (2005). RalA and RalB: Antagonistic relatives in cancer cell migration. Cancer Res..

[57] Wu Z., Owens C., Chandra N., Popovic K., Conaway M., Theodorescu D. (2010). RalBP1 is necessary for metastasis of human cancer cell lines. Neoplasia.

[58] Billhaq D.H., Lee S. (2019). A potential function of RLIP76 in the ovarian corpus luteum. J. Ovarian Res..

[59] Min J.N., Huang L., Zimonjic D.B., Moskophidis D., Mivechi N.F. (2007). Selective suppression of lymphomas by functional loss of Hsf1 in a p53-deficient mouse model for spontaneous tumors. Oncogene.

[60] Knudson C.M., Johnson G.M., Lin Y., Korsmeyer S.J. (2001). Bax accelerates tumorigenesis in p53-deficient mice. Cancer Res..

[61] Cranston A., Bocker T., Reitmair A., Palazzo J., Wilson T., Mak T., Fishel R. (1997). Female embryonic lethality in mice nullizygous for both Msh2 and p53. Nat. Genet..

[62] Embree-Ku M., Boekelheide K. (2002). FasL deficiency enhances the development of tumors in p53+/− mice. Toxicol. Pathol..

[63] Mao J.H., Perez-Losada J., Wu D., Delrosario R., Tsunematsu R., Nakayama K.I., Brown K., Bryson S., Balmain A. (2004). Fbxw7/Cdc4 is a p53-dependent, haploinsufficient tumour suppressor gene. Nature.

[64] Sansom O.J., Griffiths D.F.R., Reed K.R., Winton D.J., Clarke A.R. (2005). Apc deficiency predisposes to renal carcinoma in the mouse. Oncogene.

[65] Ling-Ling E., Zhao Y.S., Guo X.M., Wang C.Y., Jiang H., Li J., Duan C.M., Song Y. (2006). Enrichment of cardiomyocytes derived from mouse embryonic stem cells. J. Heart Lung Transplant. Off. Publ. Int. Soc. Heart Transplant..

[66] Barac A., Murtagh G., Carver J.R., Chen M.H., Freeman A.M., Herrmann J., Iliescu C., Ky B., Mayer E.L., Okwuosa T.M. (2015). Cardiovascular Health of Patients with Cancer and Cancer SurvivorsA Roadmap to the Next Level. J. Am. Coll. Cardiol..

[67] DelBove J., Kuwahara Y., Mora-Blanco E.L., Godfrey V., Funkhouser W.K., Fletcher C.D., Van Dyke T., Roberts C.W., Weissman B.E. (2009). Inactivation of SNF5 cooperates with p53 loss to accelerate tumor formation in Snf5(+/−);p53(+/−) mice. Mol. Carcinog..

[68] Palacios G., Talos F., Nemajerova A., Moll U.M., Petrenko O. (2008). E2F1 plays a direct role in Rb stabilization and p53-independent tumor suppression. Cell Cycle.

[69] Iskander K., Barrios R.J., Jaiswal A.K. (2008). Disruption of NAD(P)H:quinone oxidoreductase 1 gene in mice leads to radiation-induced myeloproliferative disease. Cancer Res..

[70] Ayrault O., Zindy F., Rehg J., Sherr C.J., Roussel M.F. (2009). Two tumor suppressors, p27(Kip1) and Patched-1, collaborate to prevent medulloblastoma. Mol. Cancer Res. MCR.

[71] Kim H.J., Barajas B., Wang M., Nel A.E. (2008). Nrf2 activation by sulforaphane restores the age-related decrease of T(H)1 immunity: Role of dendritic cells. J. Allergy Clin. Immunol..

[72] Beal M.F. (2009). Therapeutic approaches to mitochondrial dysfunction in Parkinson’s disease. Parkinsonism Relat. Disord..

[73] Damo L.A., Snyder P.W., Franklin D.S. (2005). Tumorigenesis in p27/p53- and p18/p53-double null mice: Functional collaboration between the pRb and p53 pathways. Mol. Carcinog..

[74] Greten F.R., Wagner M., Weber C.K., Zechner U., Adler G., Schmid R.M. (2001). TGF alpha transgenic mice. A model of pancreatic cancer development. Pancreatology.

[75] Shing D.C., Trubia M., Marchesi F., Radaelli E., Belloni E., Tapinassi C., Scanziani E., Mecucci C., Crescenzi B., Lahortiga I. (2007). Overexpression of sPRDM16 coupled with loss of p53 induces myeloid leukemias in mice. J. Clin. Investig..

[76] McIlhatton M.A., Murnan K., Carson D., Boivin G.P., Croce C.M., Groden J. (2015). Genetic Manipulation of Homologous Recombination In Vivo Attenuates Intestinal Tumorigenesis. Cancer Prev. Res..

[77] Prince T.L., Lang B.J., Guerrero-Gimenez M.E., Fernandez-Muñoz J.M., Ackerman A., Calderwood S.K. (2020). HSF1: Primary Factor in Molecular Chaperone Expression and a Major Contributor to Cancer Morbidity. Cells.

[78] Hu Y., Mivechi N.F. (2003). HSF-1 interacts with Ral-binding protein 1 in a stress-responsive, multiprotein complex with HSP90 in vivo. J. Biol. Chem..

[79] Samuel V.T., Shulman G.I. (2012). Mechanisms for insulin resistance: Common threads and missing links. Cell.

[80] Yaghootkar H., Scott R.A., White C.C., Zhang W., Speliotes E., Munroe P.B., Ehret G.B., Bis J.C., Fox C.S., Walker M. (2014). Genetic evidence for a normal-weight “metabolically obese” phenotype linking insulin resistance, hypertension, coronary artery disease, and type 2 diabetes. Diabetes.

[81] Singhal J., Nagaprashantha L., Vatsyayan R., Awasthi S., Singhal S.S. (2011). RLIP76, a glutathione-conjugate transporter, plays a major role in the pathogenesis of metabolic syndrome. PLoS ONE.

